# Risk Factors Contributing to Hepatitis C Virus Infection in Kashmir: A Meta-Analysis

**DOI:** 10.7759/cureus.85382

**Published:** 2025-06-05

**Authors:** Sana R Khuroo, Heena Nazir, Yatin Talwar, Jaspinder Pratap Singh, Preeti Chowdhary, Ramandeep Kaur, Umar R Khan, Abid Manzoor

**Affiliations:** 1 Community Medicine, Shri Mata Vaishno Devi Institute of Medical Excellence (SMVDIME), Katra, IND; 2 Community Medicine, Sher-i-Kashmir Institute of Medical Sciences (SKIMS), Srinagar, IND; 3 Hospital Administration, Shri Mata Vaishno Devi Institute of Medical Excellence (SMVDIME), Katra, IND; 4 Forensic Medicine, Shri Mata Vaishno Devi Institute of Medical Excellence (SMVDIME), Katra, IND; 5 Obstetrics and Gynaecology, Kalpana Chawla Medical College and Hospital, Karnal, IND; 6 Medicine, Heartlands Hospital, Birmingham, GBR; 7 Microbiology, Government Medical College Handwara, Handwara, IND; 8 Physiology, Shri Mata Vaishno Devi Institute of Medical Excellence (SMVDIME), Katra, IND

**Keywords:** hepatitis c virus, kashmir, meta-analysis, public health strategies, risk factors, unsafe medical practices

## Abstract

Hepatitis C virus (HCV) infection is a growing public health concern in Kashmir. This meta-analysis identifies and quantifies key risk factors associated with HCV in the region to guide public health interventions. A systematic review of six studies conducted in Jammu and Kashmir analyzed data from various population groups. The overall HCV prevalence in a general population survey was 38.37%, with injection drug use and dental procedures identified as major risk factors. Among hospitalized jaundiced children, 2% tested HCV-positive, both linked to chronic liver disease. A large retrospective study on blood donors (n = 97,427) found a low HCV prevalence (0.20%), with no cases among repeat voluntary donors, reinforcing the safety of voluntary blood donation. High-risk groups, including intravenous drug users (42.16%) and people who inject drugs (PWIDs) (10%), exhibited significantly higher HCV prevalence. Most PWIDs were young (69% aged 16-25 years) and from urban areas (73.5%). Financial constraints hindered treatment access for 17% of infected individuals. A molecular epidemiology study in Jammu reported 8.33% HCV prevalence, with genotype 3 being the most common strain. This meta-analysis highlights unsafe medical practices, intravenous drug use, and unregulated body modifications as major contributors to HCV transmission in Kashmir. Urgent interventions, including harm reduction programs, improved healthcare practices, and public awareness campaigns, are necessary to curb HCV spread in the region. Strengthening healthcare infrastructure and ensuring accessible treatment can significantly reduce HCV-related morbidity and mortality in Kashmir.

## Introduction and background

Hepatitis C virus (HCV) infection is a significant global health concern, affecting an estimated 58 million people worldwide, with about 1.5 million new cases annually [1]. Its chronic nature often progresses to serious liver complications such as cirrhosis and hepatocellular carcinoma. Despite major advances in antiviral therapies that have improved outcomes, prevention of HCV transmission remains a challenge, especially in resource-limited and high-prevalence regions. The Kashmir Valley is one such hotspot, where geographic, economic, and sociopolitical factors intensify the burden of infection [2]. HCV is mainly transmitted through contact with infected blood, most commonly due to unsafe medical practices, intravenous drug use, and unregulated body modifications like tattooing and piercing. The infection often remains undetected for years, contributing to its designation as a “silent epidemic.” In Kashmir, limited healthcare infrastructure, conflict-related disruptions, and cultural practices have amplified transmission risks [3]. Addressing this requires targeted public health interventions aligned with global goals, including the WHO’s aim to eliminate HCV as a public health threat by 2030 [4].

HCV in Kashmir and rationale of the study

The Kashmir Valley faces unique challenges in HCV control due to its geographical, economic, and sociopolitical landscape [5]. Over the past decade, the region has witnessed a rise in HCV prevalence, often exceeding national averages, driven by unsafe medical practices such as the reuse of needles and syringes, unregulated blood transfusions, and inadequate screening protocols [6-8]. Intravenous drug use and unsterile body modification procedures further exacerbate the transmission risk [7-10]. Conflict-related disruptions and limited healthcare infrastructure have heightened the population’s vulnerability to HCV and other infectious diseases [5,10]. This trend aligns with patterns observed in low- and middle-income countries, stressing the need for region-specific public health interventions [11]. Successful strategies from other regions, such as harm reduction, blood safety programs, and awareness campaigns, offer actionable models for Kashmir [12]. A comprehensive understanding of these risk factors and regional dynamics is vital to guide effective control measures and contribute to global HCV elimination goals [13,14]. Despite research on HCV, gaps remain in understanding the specific risk factors and drivers in the Kashmir Valley [15]. Many studies focus on global or national trends, overlooking the region’s unique challenges. The rising prevalence of HCV in Kashmir highlights the need for region-specific insights to inform public health strategies [16]. Variations in study methodologies and inconsistent findings have hindered a clear understanding of key risk factors. This study aims to fill these gaps by synthesizing available evidence using a meta-analytic approach, providing robust, context-specific estimates. The study supports the global goal to eliminate HCV by 2030, emphasizing the need for targeted interventions in Kashmir, where limited healthcare and low awareness make addressing key risk factors crucial to breaking the transmission cycle. The findings will guide prevention strategies and contribute to improving the health of affected communities [16].

## Review

Materials and methods

A comprehensive literature search was conducted across major electronic databases, including PubMed, Scopus, Web of Science, Google Scholar, and Embase, to identify relevant studies published up to 2024. The search strategy included peer-reviewed articles written in English, as well as studies in other languages when reliable translations were available. Keywords and Medical Subject Headings (MeSH) such as “hepatitis C virus”, “Kashmir”, “risk factors”, and “epidemiology” were used, utilizing Boolean operators (AND/OR) and synonymous terms to ensure a broad and inclusive search. In addition to database queries, the reference lists of all selected articles and relevant systematic reviews were reviewed to identify any additional eligible studies that may have been missed. Studies were considered eligible for inclusion if they met the following criteria: conducted in the Kashmir region, reported on risk factors associated with HCV infection, used a cross-sectional, case-control, or cohort study design, and were published in English in peer-reviewed journals. Studies were excluded if they presented incomplete or missing data, review articles, case reports, conference abstracts, or focused on regions outside of Kashmir or on diseases other than HCV.

Data extraction and quality assessment

Two independent reviewers screened the titles and abstracts for eligibility. Full-text articles of potentially relevant studies were then assessed for inclusion. Data extraction was performed using a standardized form, capturing details on study design, sample size, population characteristics, reported risk factors, and outcomes related to HCV infection. Discrepancies between reviewers were resolved through discussion or consultation with a third reviewer. Quality assessment was conducted using broad critical evaluation guidelines. Selected studies were subjected to a rigorous quality evaluation to assess heterogeneity and determine the appropriateness of including studies in the meta-analysis. A comprehensive evaluation technique was employed, focusing on the PICO (Population, Intervention, Comparison, and Outcome) criteria.

Data collection strategies

**Figure 1:**
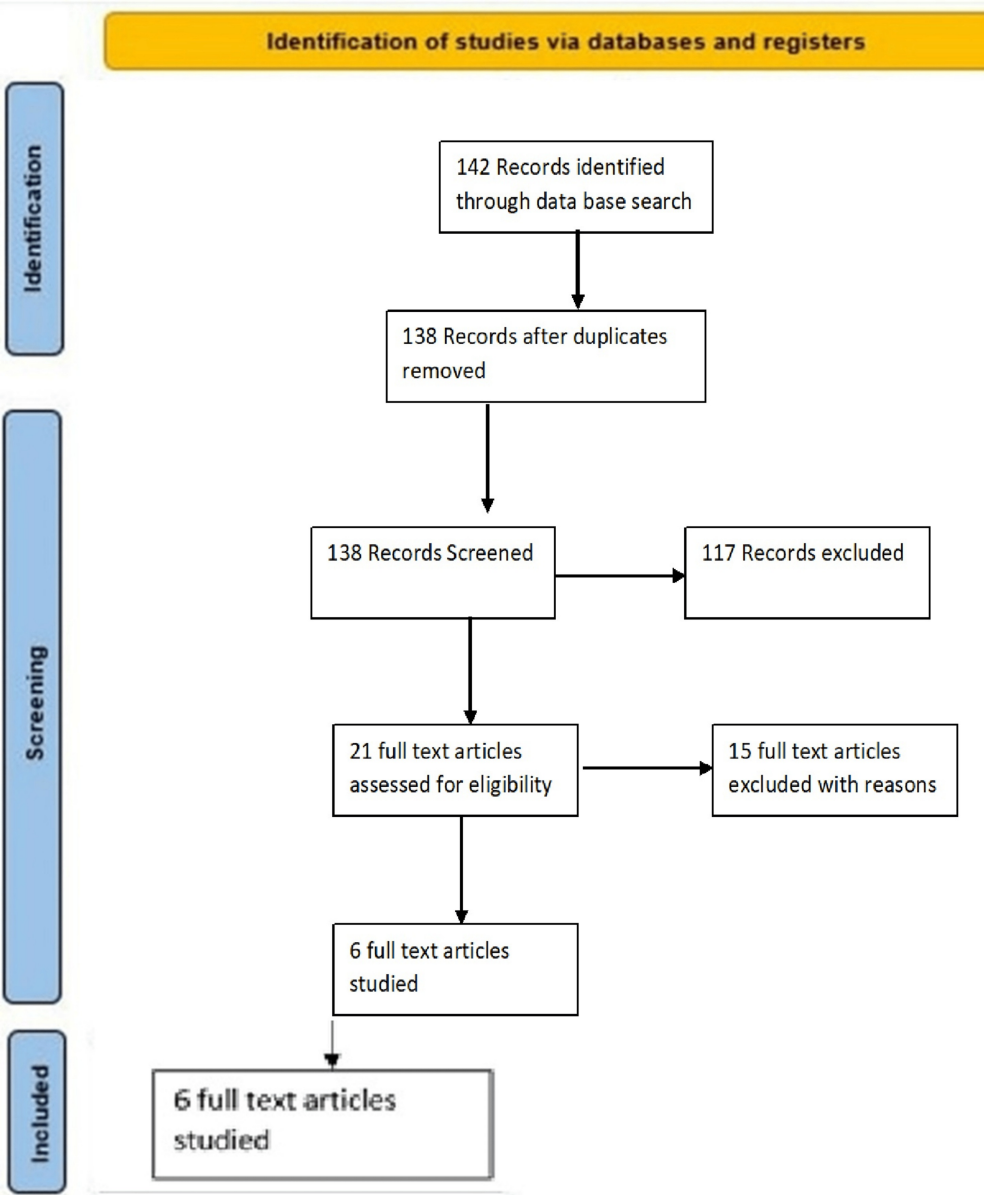
PRISMA 2020 Flow Diagram PRISMA, Preferred Reporting Items for Systematic Reviews and Meta-Analyses

To begin the systematic review and meta-analysis, databases such as Scopus, PubMed, Web of Science (ISI), and Google Scholar were searched. The keywords “hepatitis C virus”, “Kashmir”, “risk factors”, and “epidemiology” were used, as well as all possible combinations of these terms. No time limits were applied in the search process, and the metadata from identified studies were transferred into EndNote reference management software. To ensure comprehensiveness, the reference lists of all collected articles were manually reviewed. The inclusion and exclusion criteria were developed using the PICO framework and are summarized as follows: Population (P): individuals in the Kashmir region at risk of or affected by HCV infection; Interest (I): risk factors contributing to HCV infection (e.g., unsafe medical practices, intravenous drug use); Context (Co): sociocultural, healthcare, and epidemiological conditions in the Kashmir Valley. Ideally, studies included were those that investigated the determinants and prevalence of HCV in this population with an emphasis on contributing risk factors. In an effort to limit the search results to manageable levels, studies older than 10 years were excluded. Non-English articles were also excluded due to potential language bias and issues with translation accuracy. The search was initially conducted using Boolean operators, followed by filtering based on the inclusion criteria. This process resulted in 28 articles from Google Scholar, 39 from Medline, and 75 from PubMed. From the 142 articles identified, a PRISMA flow diagram (Figure 1) was used to determine the selection process. Several studies were excluded as they did not align with the research question. Duplicates were removed, and abstracts were reviewed. Finally, six articles met the inclusion criteria and were included in the meta-analysis. The authors utilized the Newcastle-Ottawa Scale (NOS), which is a widely accepted tool for assessing the quality of non-randomized studies in meta-analyses.

Results

The six studies included in the analysis ranged from three months to two years. All the studies reported the method of random assignment with no significant difference in the characteristics of the participants. Table 1 displays an overview of each article.

**Table 1:** Key Findings From Hepatitis C Virus (HCV) Studies in Kashmir BST-HCV, specific questionnaire that is used to evaluate the knowledge, attitudes, and practices of patients with Hepatitis C virus infection, particularly focusing on treatment adherence; EIA, enzyme immunoassay; ELISA, enzyme-linked immunosorbent assay; FHF, fulminant hepatic failure; GMC, Government Medical College; HBsAg, hepatitis B surface antigen; IDUs, injecting drug users; KAP, knowledge, attitudes, and practices; PWIDs, people who inject drugs; SKIMS, Sher-i-Kashmir Institute of Medical Sciences

Author (year)	Sample/setting	Method	Key findings
Ahmed et al., 2018 [17]	100 hospitalized jaundiced children, GMC, Srinagar	Hospital-based prospective; ELISA + PCR - oral questionnaire on HCV infection aspects - PCR confirmation for positive cases	HCV: 2%; hepatitis A: 74%; two HCV+ had CLD; one HBsAg+; eight FHF cases. Emphasizes screening and awareness.
Qureshi et al., 2016 [18]	97,427 blood donors, SKIMS (2003-2012)	Retrospective; seropositivity testing	HBV/HCV lower than national average; voluntary donors safer. Promotes voluntary donation.
Rehman et al., 2016 [5]	2051 village residents	Community survey; rapid test + EIA	Unsafe injections major HCV risk. Urges sterilized equipment, quack regulation, public education.
Wani et al., 2021 [19]	200 PWIDs, tertiary hospital	Prospective (2017-2020); standard testing	High HBV/HCV among PWIDs. Calls for awareness, screening, and better care access.
Chowhan, 2019 [20]	268 IDUs, private de-addiction clinic, Jammu	Cross-sectional; questionnaires (KAP, BST-HCV)	High HCV in IDUs; low treatment due to awareness, stigma, cost. Calls for free testing/treatment.
Sharma et al., 2017 [21]	396 patients, GMC Jammu	Observational; ELISA + genotyping	Shows HCV genotypes in region. Recommends larger studies to guide regional treatment strategies.

**Figure 2:**
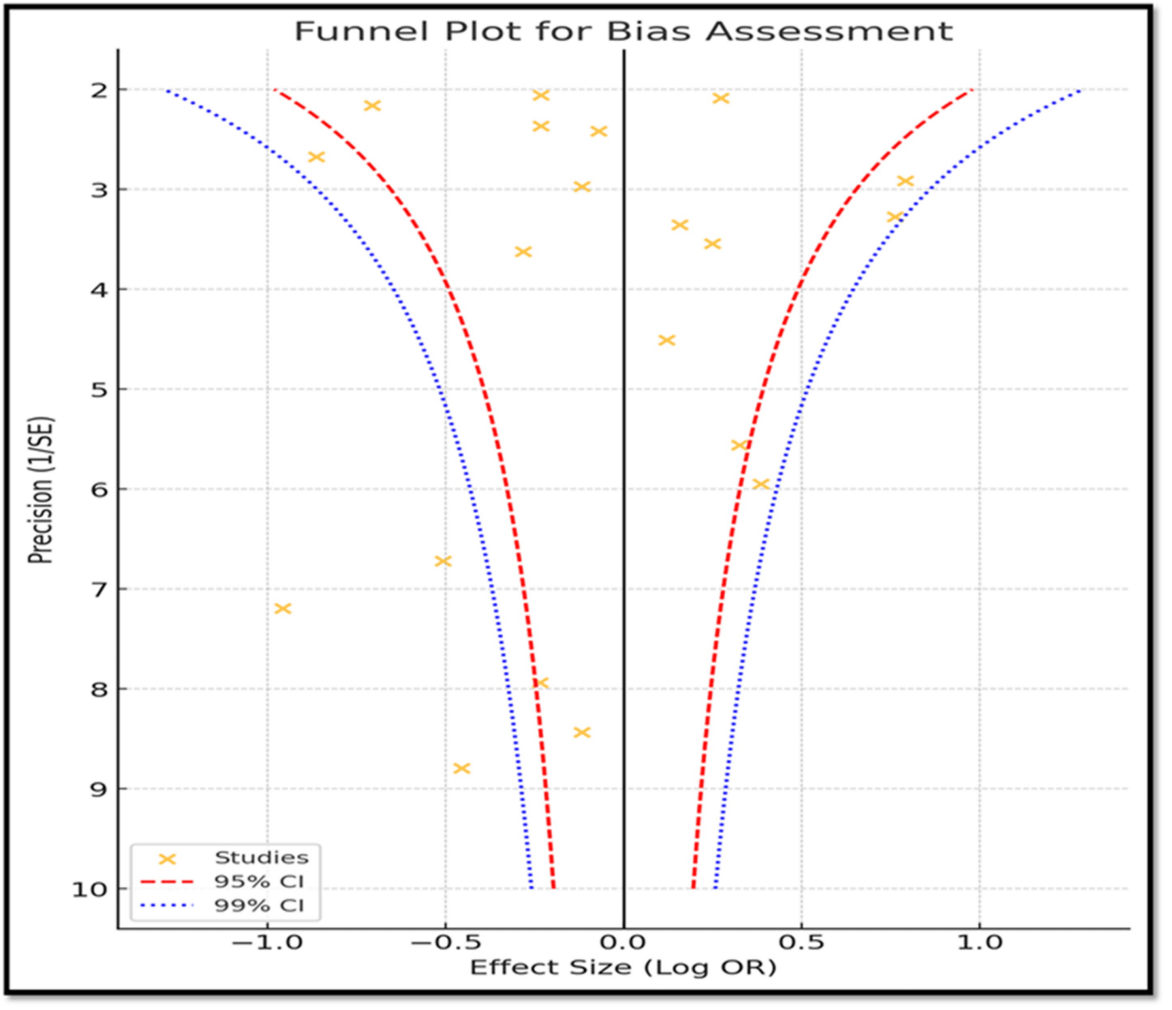
Funnel Diagram Showing Funnel Plot for Publication Bias Assessment

The funnel plot (Figure 2) appears symmetric, indicating low publication bias. Studies cluster around the mean effect size, with higher precision studies near the top and lower precision studies at the bottom, as expected in an unbiased meta-analysis. Most studies fall within the 95% and 99% confidence intervals, suggesting no significant small-study effects or selective reporting bias. The balanced distribution on both sides of the null effect line further confirms minimal publication bias.

Discussion

Hepatitis B and C viruses continue to pose significant public health challenges, particularly among high-risk populations such as intravenous drug users, hospitalized children, and blood donors. The studies reviewed in this analysis shed light on the epidemiology of HCV in Kashmir, highlighting varying prevalence rates and associated risk factors. Ahmed et al. [17] conducted a hospital-based study on jaundiced children and found a low prevalence of HCV (2%), with all positive cases linked to chronic liver disease. This suggests that pediatric HCV often remains undiagnosed until more advanced stages. In contrast, Qureshi et al. [18] examined blood donors over a 10-year period and found a low overall prevalence of HCV (0.20%), with replacement donors exhibiting higher infection rates than voluntary donors. This underscores the importance of promoting voluntary blood donations for safer transfusion practices.

A more alarming finding came from the study by Rehman [5], which reported a high prevalence of HCV (38.37%) in the general population, particularly linked to unsafe medical practices, such as unsterile injections and dental procedures. This study highlighted the need for urgent interventions, including improved infection control in medical and dental settings. Similarly, Wani et al. [19] and Chowhan and Sakral [20] focused on people who inject drugs (PWIDs) and intravenous drug users, respectively, and found alarmingly high rates of HCV prevalence (10% and 42.16%), with younger and urban populations most affected. Financial constraints were identified as a major barrier to treatment for 17% of HCV-positive drug users, reinforcing the need for affordable healthcare options. Sharma et al. [21] provided valuable insights into the molecular epidemiology of HCV in Jammu, finding genotype 3 to be the most prevalent strain. This aligns with national data and suggests that targeted treatment strategies based on HCV genotype could improve outcomes. Collectively, these studies illustrate that while the general blood donor population shows relatively low seropositivity rates, certain high-risk groups, such as intravenous drug users and individuals exposed to unsafe medical practices, experience significantly higher HCV prevalence. The variation in prevalence rates across different populations emphasizes the need for targeted, context-specific public health strategies. These findings highlight the urgent need for expanded screening programs, increased access to antiviral therapies, and robust harm reduction initiatives to prevent further transmission of HCV and HBV. In addition to medical interventions, educational campaigns aimed at healthcare workers and the general population are critical in reducing risk factors such as unsafe injection practices, inadequate sterilization of medical equipment, and unregulated medical procedures. Public health interventions that address these issues could significantly reduce the burden of viral hepatitis in Kashmir and other similar endemic regions. Table 2 presents a comparative overview of key findings from multiple studies on the prevalence of HCV in Kashmir. The studies include diverse populations, such as hospitalized children, blood donors, intravenous drug users, and the general community, providing a comprehensive understanding of HCV epidemiology in the region.

**Table 2:** HCV Prevalence in Kashmir - Summary CLD, chronic liver disease; HCV, hepatitis C virus; IDU, injecting drug users; PWIDs, people who inject drugs; SKIMS, Sher-i-Kashmir Institute of Medical Sciences

Study	Study type	Population	Sample size (n)	HCV prevalence (%)	Key findings
Ahmed et al., 2018 [17]	Prospective hospital-based	Jaundiced children, Kashmir	100	2.0	HCV low; all HCV + had CLD; hepatitis A: 74%.
Qureshi et al., 2016 [18]	Retrospective	Blood donors at SKIMS, Kashmir	97,427	0.20	Higher in replacement donors; 0% in repeat donors; trend declining.
Rehman et al., 2016 [5]	Community-based survey	General population, Kashmir	2,051	38.37	High rate; linked to injections/dental work; genotype 3a dominant.
Wani et al., 2021 [19]	Prospective descriptive	PWIDs	200	10.0	High burden; 73.5% urban; 69% aged 16-25.
Chowhan, 2019 [20]	Cross-sectional	IDUs, de-addiction clinic	268	42.16	High in youth, unmarried, poor; 17% untreated due to cost.
Sharma et al., 2017 [21]	Molecular epidemiology study	HCV-positive, Jammu	396	8.33	Genotype 3 common; follows national pattern.

Summary of key findings

The meta-analysis reveals significant variation in HCV prevalence across different population groups in Kashmir, ranging from 0.20% in blood donors to 42.16% in intravenous drug users. Notably, unsafe medical practices, including unsterile injections and dental procedures, were commonly associated with HCV transmission. Genotype 3a emerged as the predominant strain in most studies, indicating the need for genotype-specific treatment strategies. Studies also highlighted the demographic characteristics of those most affected, with younger, unmarried individuals and those from lower socioeconomic backgrounds showing higher infection rates. Public health interventions should be tailored to these groups, with a focus on improving healthcare safety, expanding harm reduction programs, and enhancing access to treatment. The findings emphasize the urgency for more comprehensive screening programs, increased awareness of HCV risk factors, and better access to antiviral therapies, especially for high-risk groups. Additionally, the relatively high prevalence of asymptomatic cases calls for more rigorous screening to detect and treat infections at an earlier stage.

**Figure 3:**
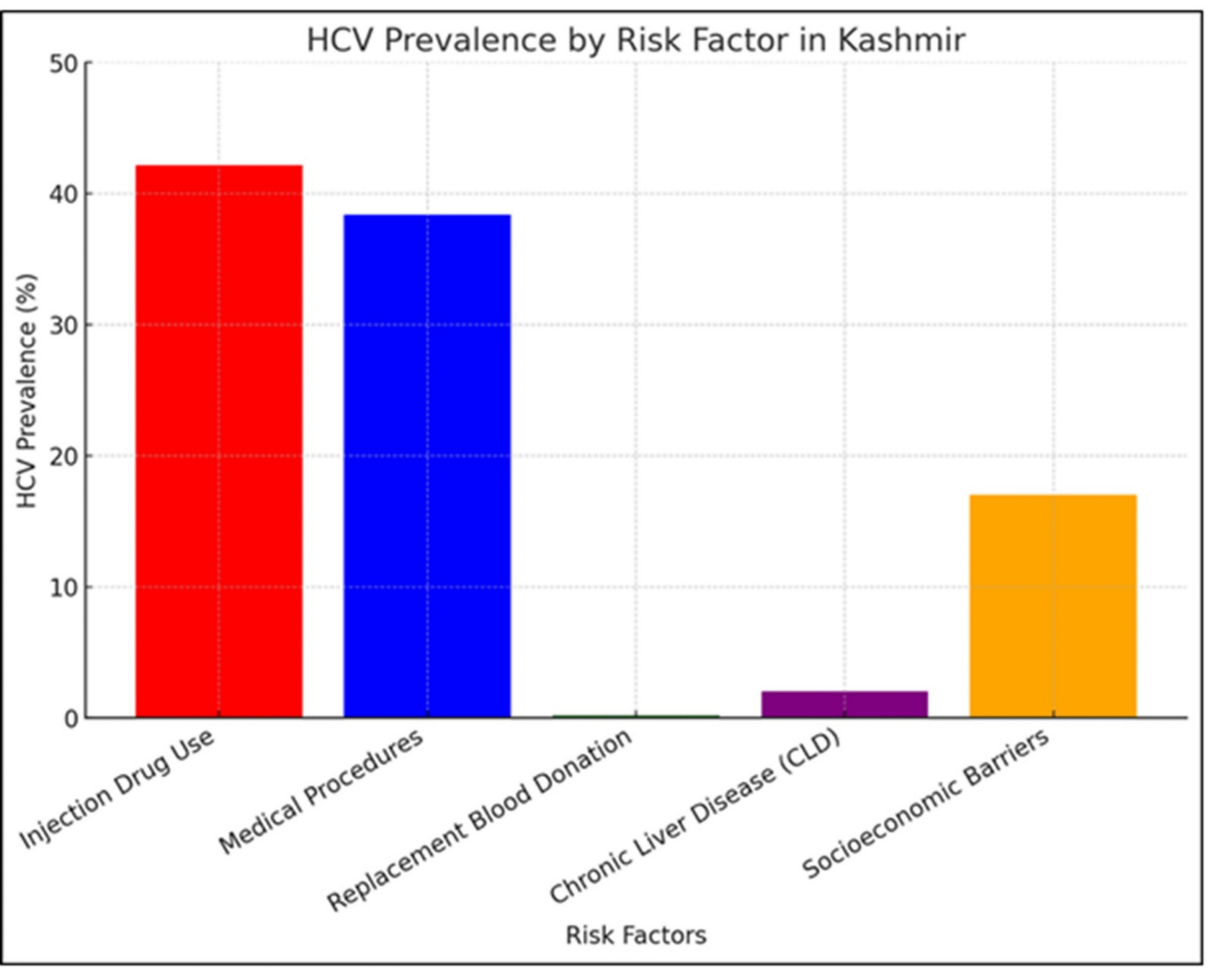
Risk Factor Prevalence Across Six Studies HCV, hepatitis C virus

Figure 3 shows HCV prevalence across various risk factors in Kashmir. Injection drug users (42.16%) have the highest prevalence, followed by those undergoing medical procedures (38.37%) and individuals facing socioeconomic barriers (17%). Chronic liver disease was observed in all HCV-positive jaundiced children (2.0%), while voluntary blood donation had the lowest prevalence (0.20%). These findings highlight the need for targeted interventions, harm reduction, and better healthcare practices to reduce HCV transmission.

Bias assessment

The bias assessment of this meta-analysis indicates minimal publication bias, as the funnel plot shows a symmetrical distribution of studies. Most studies fall within expected confidence intervals, suggesting a balanced representation of findings. However, selective reporting in smaller studies and factors, such as reliance on published literature, exclusion of non-English studies, and variations in study methods, could introduce some bias. Additionally, differences in sample populations and diagnostic approaches may limit the generalizability of the results.

## Conclusions

This meta-analysis highlights the significant burden of HCV in Kashmir, particularly among high-risk groups, such as intravenous drug users (42.16%) and those undergoing unsafe medical procedures (38.37%). The findings emphasize the need for targeted public health interventions, including harm reduction, improved infection control, and better access to treatment. Addressing financial barriers and raising awareness are key to reducing transmission and improving health outcomes in the region.
